# Bumble Bee Probability of Occurrence Responds to Interactions Between Local and Landscape Land Use, Climatic Niche Properties and Climate Change

**DOI:** 10.1111/ele.70145

**Published:** 2025-05-27

**Authors:** Tim Newbold, Jeremy Kerr, Peter Soroye, Jessica J. Williams

**Affiliations:** ^1^ Centre for Biodiversity and Environment Research, Department of Genetics, Evolution and Environment University College London London UK; ^2^ Department of Biology University of Ottawa Ottawa Ontario Canada

**Keywords:** bumble bees, climate change, fertiliser, land use, natural habitat, pesticide, pressure interactions, probability of occurrence, thermal niche

## Abstract

Insect biodiversity is changing rapidly, driven by a suite of pressures, notably land use, land‐use intensification and increasingly climate change. We lack large‐scale evidence on how land use and climate change interact to drive insect biodiversity changes. We assess bumble bee responses to interactive effects of land use and climate pressures across North America and Europe. The probability of occurrence increases in landscapes with a higher proportion of natural habitat and a shorter history of human disturbance. Responses to climate warming relative to historical conditions are weakly negative in natural habitats but positive in human land uses, while human land use reduces the probability of occurrence most in the centre of species' temperature niches. We estimate that the combined pressures have reduced bumble bee probability of occurrence by 44% across sampled natural habitats and 55% across human land uses, highlighting the pervasive influence that human pressures have had on biodiversity across habitats.

## Graphical abstract

We analyse a collation of bumble bee presence and absence data from across the continents of Western Europe and North America, spanning both natural and human‐modified habitats. We find that bumble bee probability of occurrence increases in landscapes with a higher proportion of natural habitat and a shorter history of human disturbance. Responses to climate warming relative to historical conditions are weakly negative in natural habitats but positive in human land‐use types, while human land use reduces probability of occurrence most in the centre of species' temperature niches.

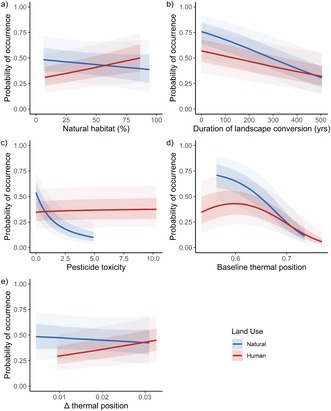

## 1 Introduction

Insect communities are changing rapidly, although reported biodiversity trends differ in direction and magnitude. Several studies have detected steep declines in the abundance, species richness and distributions of terrestrial insects (Janousek et al. 2023; Kerr et al. 2015; van Klink et al. 2020; Lister and Garcia 2018), while other studies report little overall change in insect biodiversity on average (Macgregor et al. 2019; Outhwaite et al. 2020).

Human land use and land‐use intensification are key drivers of insect biodiversity changes. For bees, conversion of natural habitats to agriculture and other human uses often has negative effects (De Palma et al. 2017; Evans et al. 2018; Janousek et al. 2023; Samuelson et al. 2018), although responses vary strongly among species (Cariveau et al. 2013; Prestele et al. 2021) and regions (De Palma et al. 2016). Within agricultural areas, intensification of farming practices (e.g., lowering of crop diversity, or removal of flower‐rich field margins) is associated with further reductions, on average (Hemberger et al. 2021; Kennedy et al. 2013). One facet of agricultural intensification that is particularly consequential for bees is the application of chemical pesticides, with toxicity to bees often increasing over time (Douglas et al. 2020), leading to reductions in bee distributions, population persistence, and reproductive performance (Nicholson et al. 2024; Siviter et al. 2021; Stuligross and Williams 2021; Whitehorn et al. 2012; Woodcock et al. 2016).

The biodiversity impacts of habitat modifications can operate over wider landscapes and longer timescales than captured by localised biodiversity samples. Natural habitat availability in the landscape is important for maintaining bee diversity, including in agricultural habitats (Evans et al. 2018; Gutiérrez‐Chacón et al. 2018; Mola et al. 2021; Proesmans et al. 2019; Rutschmann et al. 2022). However, responses often vary among species and types of natural habitat (e.g., forest versus grassland; Goulson et al. 2010; Gutiérrez‐Chacón et al. 2018; Kammerer et al. 2021), and are sometimes absent or negative, for example when farmed areas provide abundant floral resources (Christman et al. 2022; Mola et al. 2021). Farming practices may impact biodiversity within adjacent natural habitats, for example, through drift of pesticides and other agricultural chemicals out of the farmlands to which they are applied (Krupke et al. 2012). Over time, agricultural intensity often increases, with loss of remnant natural habitats (Tscharntke et al. 2005), effects which are associated with reductions in bumble bee occurrence (Hemberger et al. 2021).

Climate change exerts a strong influence on bees and other insects, and its effects are growing rapidly. Already, bumble bee distributions have been contracting at southern range margins (Kerr et al. 2015) and moving to higher elevations (Pyke et al. 2016), while the Community Temperature Index of communities (the average temperature affiliation of species in a community) has been increasing (Fourcade et al. 2019; Hemberger and Williams 2024), consistent with expectations under climate warming. Indeed, analyses of temporal changes in bee diversity have revealed an important role for warming temperatures in observed declines (Janousek et al. 2023; Kammerer et al. 2021; Soroye et al. 2020). Future projections suggest that climate change impacts on bumble bees are likely to increase (Marshall et al. 2018; Prestele et al. 2021).

Recent evidence points toward interactive effects of land use, land‐use intensification and climate change on biodiversity, but fewer studies have focused on insects than on the better‐studied vertebrates (e.g., Fourcade et al. 2019; Hamblin et al. 2017; Janousek et al. 2023; Kammerer et al. 2021; Oliver et al. 2017; Outhwaite et al. 2022; Waldock et al. 2020). Such interactive effects are driven by two key mechanisms: habitat disturbance impeding species' range shifts in response to climate change (Oliver and Morecroft 2014); and local climatic changes associated with conversion of natural habitats (Oliver and Morecroft 2014; Williams and Newbold 2020). Insect species tolerant of warmer and drier conditions are, on average, more likely to occur within agricultural and urban land uses than other species (Hamblin et al. 2017; Oliver et al. 2017; Waldock et al. 2020), probably resulting from altered local climatic conditions in these areas. Studies on vertebrates have revealed that populations living close to the upper realised thermal niche limit of species respond more negatively to human land uses than other populations (Williams and Newbold 2021).

We present a continental‐scale study, encompassing Europe and North America, considering the interacting effects of climate change, land use and land‐use intensification on the average occurrence probability of bumble bees. A number of studies have investigated large‐scale (nation or continent‐wide) effects of climate and land‐use variables on bees. Notably, Fourcade et al. (2019) assessed change in bumble bee species richness in Norway as a result of climate and land‐cover changes, Kammerer et al. (2021) tested the effect on bee species richness of climate, land‐cover and insecticide toxic load in three US states, Janousek et al. (2023) showed climate change, land cover and pesticide application impacts on the western bumble bee across the US, and Hemberger and Williams (2024) documented restructuring of bumble bee assemblages in the US favouring species tolerant of higher temperatures. Here, we make three further developments over previous work. First, we use a recent characterisation of the realised thermal niche of bumble bees (Soroye et al. 2020) to test the effect of climate change dependent upon the position of a population within species' realised thermal niche, allowing us to go beyond recent work assessing general interactive effects of land use and climate change across all insects (Outhwaite et al. 2022). Second, we use estimates of land‐use history to test whether bumble bee occurrence depends on the time since landscapes were converted to human use, in conjunction with present land uses, habitat extent, and pesticide toxicity. We expect that bumble bee occurrence probability will be lower in agricultural areas, particularly where land‐use intensity is higher, where landscapes have been dominated by human activities for longer, and where less natural habitat remains within the landscape. We further predict that occurrence probability will decline most strongly in agricultural land uses where bumble bee populations are near their upper thermal limits, and especially where temperature has recently increased. Third, we use the models to infer how bumble bee occurrence probability within sampled habitats (both natural and human‐modified habitats) has been shaped by the pressures currently acting on them.

## 2 Material and Methods

### 2.1 Data on Responses to Land Use

Data describing bumble bee occurrence (recorded presences and absences) across land‐use types were derived from the PREDICTS database, which collates biodiversity records from individual studies that conducted snapshot spatial samples of biodiversity along land‐use or land‐use‐intensity gradients (Hudson et al. 2017). Studies included in the database had to meet four criteria: (1) sampling methods were published; (2) biodiversity was sampled at more than one location; (3) sampled locations spanned a gradient of land‐use type and/or intensity and (4) the sampling protocol was the same across sampled sites. Any records without geographical coordinates were omitted from our analysis.

The final dataset was derived from 53 original studies (listed in the Supporting Information) and consisted of 17,966 records for 49 bumble bee species, sampled at 1675 locations in 13 countries in North America and Europe (Figure S1; Table S1). The original samples collated in the PREDICTS database were recorded in the field between 2000 and 2011. Sampled species were those assessed as being of lower risk of extinction (at least for European species; Figure S2), which are expected to respond less to environmental changes, thus likely rendering inferred effects of climate change and land use conservative.

### 2.2 Explanatory Variables

We considered local land use by dividing sampled sites into natural or human‐modified habitats. In the PREDICTS database, land‐use type is classified, based on the habitat descriptions provided by the original authors, into six broad categories: primary vegetation (natural habitat with no record of past destruction), secondary vegetation (habitat recovering to its natural state after past destruction by human actions or extreme natural events), plantation forests (areas used to cultivate woody crops), cropland (areas used to cultivate herbaceous crops, including fodder for livestock), pasture (areas used regularly or permanently to graze livestock), and urban (areas of human settlement, buildings, or managed for recreation). We treated primary and secondary vegetation as natural habitats, and all other land‐use types as human‐modified. Although bumble bees may respond more strongly to landscape habitat availability than to local land‐use conditions (Janousek et al. 2023), the latter can be important (Evans et al. 2018) and are considered here also because they are fundamental to the sampling structure of the PREDICTS database (Hudson et al. 2014).

We investigated how surrounding habitat conditions, cumulative pesticide toxicity, and species' thermal niche properties shape bumble bee occurrence in both natural and human‐modified habitats. Information on habitat condition in the landscapes surrounding sampled sites comprised estimates of natural habitat availability and the length of time the habitat has been substantially modified by humans. We obtained estimates of the percentage of natural habitat in the 2 × 2‐km grid cell within which biodiversity was sampled (we tested the robustness of our results to using 1‐km and 5‐km grid cells; Figures S7 and S8). Original mapped estimates of different land‐use types were from a down‐scaled land‐use projection for 2005 at 30‐arc‐second spatial resolution (Hoskins et al. 2016). These land‐use maps estimate the fraction of each grid cell covered by five out of the six land‐use types recognised in the PREDICTS database (not plantation forests) and were evaluated against the land‐use classifications in the PREDICTS database (Hoskins et al. 2016). We summed the primary and secondary vegetation maps to obtain estimates of natural habitat. We projected and resampled this map of fractional cover of natural habitats to an equal‐area grid (Behrmann projection) at 1‐km resolution, using bilinear interpolation (using the terra R package Version 1.7–71; Hijmans 2024). All data‐processing code was implemented in R Version 4.3.1 (R Core Team 2023). We then aggregated the resulting 1‐km map by factors of two or five, to obtain the maps at 2‐km and 5‐km grain, calculating the mean fractional natural‐habitat cover. Although there is a tendency for the percentage of surrounding natural habitat to be lower for human‐modified land uses compared to natural habitats, this association is weak (ANOVA *R* ^2^ = 0.06 at 2‐km grain). We derived estimates of the duration of substantial human habitat modification from the Land‐Use Harmonisation Project land‐use reconstruction at 0.5° spatial resolution (Hurtt et al. 2011), which estimates the fractional cover of primary vegetation, secondary vegetation, croplands, pastures, and urban areas from 1500 to 2005. The HYDE historical land‐use model, from which the reconstructed land‐use estimates are interpolated, has an interval of one century until 1700 and decadal thereafter (Klein Goldewijk et al. 2011). Thus, longer estimates of the duration of landscape modification are subject to a higher degree of uncertainty. We measured the duration of substantial human habitat modification as the number of years since a 0.5° grid cell was first 30% converted to human‐modified land‐use types (croplands, pastures or urban areas). Above 30% loss of natural habitats from landscapes, many of the most sensitive bird and mammal species are substantially negatively impacted (Andrén 1994). The appropriate threshold for insect species (including bumble bees) is unclear. Therefore, we tested the sensitivity of using conversion thresholds of 10% or 50% (Figures S9 and S10). Grid cells that did not reach a given conversion threshold were considered to have a substantial modification duration of 0 years.

To calculate pesticide toxicity, we used modelled estimates of application density (kg/ha) of the 20 dominant pesticide active ingredients for each of 6 individual crops (corn, soyabean, wheat, cotton, rice, and alfalfa) or 4 crop groups (vegetables and fruit, orchards and grapes, pasture and hay and other crops)—totalling 95 different active ingredients across all crops (Maggi et al. 2019). These data are presented at 5‐arc‐minutes spatial resolution. We used the low rather than high modelled estimates in our main models, but tested the robustness of our models to using the high estimates (Figure S11). Estimates of application density for each pesticide active ingredient were divided by estimates of LD50 for that chemical, derived from the Pesticide Properties Database (Lewis et al. 2016), using estimates for honey bees, since records of toxicity for bumble bees are incomplete. We used estimates of contact LD50s only (available for 87/95 active ingredients). We summed these toxicity estimates across the 20 active ingredients for each of the 10 crops/crop groups (200 toxicity maps in total).

For species' thermal niche properties, we used previously published methods and associated records of bumble bee occurrence (Soroye et al. 2020). Specifically, we estimated: (1) the position of a population within the species' realised thermal niche, by estimating how the temperatures experienced by the population relate to the realised thermal niche of the species in a baseline period before the onset of rapid climate changes (1901–1975) and (2) how changes in temperature between the baseline period and a recent period (2000–2014) have shifted the position of the population within this realised thermal niche. Realised thermal niche limits were estimated for each species as the mean of the five lowest minimum monthly temperatures and the mean of the five highest maximum monthly temperatures across all bumble bee spatial records in the baseline period (from an extensive database of species occurrence records across Europe and North America; see Soroye et al. 2020, for details). We then derived the maximum monthly temperatures for all 12 months in each baseline or recent year, and rescaled them such that a value of 0 equates to the minimum realised thermal niche limit of the species, and a value of 1 to the maximum niche limit. We then averaged the 12 (rescaled) monthly values to derive the final mean niche position for each year. Finally, we averaged estimates across all years in either the baseline or recent period. A thermal niche position value of 0 represents a population at the lower limit of the species' realised thermal niche, while a value of 1 represents a population at the upper limit of the species' realised thermal niche. Monthly minimum and maximum temperature estimates for the period 1901–2015 were obtained from the CRU global gridded climate reconstruction at 0.5° spatial resolution, Version 3.24.01 (University of East Anglia Climatic Research Unit et al. 2017), which was the most recent version of this dataset available at the time of calculation (Soroye et al. 2020). Because our study area encompasses regions well covered by weather stations, the CRU estimates of temperature for the sites in our analysis were all derived from data from a minimum of 22 weather stations at the beginning of the baseline period (January 1901), and 31 at the end of the recent period (December 2014).

To control for potential confounding effects of elevation, we included in the model estimates of elevation at 30‐arc‐second spatial resolution from WorldClim Version 2.1 (Fick and Hijmans 2017). All spatial data manipulation was carried out using the terra R package Version 1.7–71 (Hijmans 2024).

### 2.3 Statistical Analysis

All analysis code was implemented in R Version 4.3.1 (R Core Team 2023). We used a binomial Bayesian hierarchical model to fit species' presence or absence. Species were considered absent if they were not recorded but had been targeted by a particular study (i.e., they were recorded in at least one other sampled site within a study). As random effects, we fitted: (1) Study identity, to control for differences in sampling protocols and broad geographic differences; (2) Site identity nested within study, to control for site‐specific factors; and (3) Species identity, to account for variation in responses among species. We were unable to account for variations in sampling effort beyond incorporating the random effect of study identity, because the bumble bee data in the PREDICTS database were collected using many different sampling methods, with non‐comparable measures of sampling effort. Although there may be important differences in responses between the two continents, the dataset was too small to include such variation in the models. As a check of the robustness of our results, we also fit a zero‐inflated negative binomial model of relative abundance (Figure S12). Similarly, while there may be interspecific variation in responses, there was not sufficient sampling of each species to allow fitting of random slopes in the models. As fixed effects, we considered local land‐use type, surrounding natural habitat availability, the duration of substantial human modification of landscapes, cumulative pesticide toxicity, baseline position within species' thermal niche, change in thermal niche position between the baseline and recent periods, and elevation. To avoid model over‐fitting, given the small size of the dataset, we fitted linear effects for all continuous variables, except for baseline thermal niche position, for which we fit a quadratic polynomial. Physiological experiments have long established that, for any given species, performance tends to be maximal at intermediate temperatures and decline at colder and hotter temperatures (Pawar et al. 2024). Cumulative pesticide toxicity and elevation were log_e_‐transformed to deal with the right‐skewed distribution of values, first adding a value of 1 to pesticide toxicity and 2 to elevation to deal with zero and (for elevation) negative values. We considered interactions between local land‐use type and the other continuous variables. We also explored interactions with surrounding natural habitat availability, but estimated coefficients for these interactions were subject to a high degree of uncertainty, probably owing to slight correlations among the different landscape‐level variables. The structure of the final model was:

$$\begin{array}{c} \text{Occurrence}∼\text{LandUse}+\text{NaturalHabitat}+\text{PesticideToxicity}+\text{LandscapeModificationDuration}\\ +\text{poly}\left(\text{BaselineThermalNichePosition},2\right)+\text{DeltaThermalNichePosition}+\text{LandUse}:\text{NaturalHabitat}\\ +\text{LandUse}:\text{PesticideToxicity}+\text{LandUse}:\text{LandscapeModificationDuration}+\text{LandUse}:\text{poly}\left(\text{BaselineThermalNichePosition},2\right)\\ +\text{LandUse}:\text{DeltaThermalNichePosition}+\text{Elevation}+\left(1|\mathrm{SS}\right)+\left(1|\text{SSBS}\right)+\left(1|\text{TaxonName}\right) \end{array}$$

where ‘poly(BaselineThermalPosition,2)’ denotes the fitting of a quadratic polynomial term, ‘SS’ denotes study identity, and ‘SSBS’ denotes site identity (nested within study). All models were implemented in the brms R package Version 2.21.0 (Bürkner 2021). All parameters took the default, weakly informative priors. We ran 4 chains, and assessed convergence by inspection of Rhat values. Chains were run with a burn‐in of 1000 iterations and sampling period of 1000 iterations. For comparison, we also built a model containing just land use as a fixed effect. Model explanatory power was assessed using a version of *R* ^2^ values designed for Bayesian models (Gelman et al. 2019), calculated using the bayes_R2 function (brms R package). Collinearity among the explanatory variables was sufficiently low to prevent biases (Pearson's correlation coefficient, *r* < 0.55). We tested for spatial autocorrelation across all of the residuals of the final model, and in the residuals associated with each underlying study using Moran's tests, implemented in the spdep R package Version 1.2–8.

To reveal the impacts that cumulative pressures have had on bumble bee occurrence probability across natural and human‐modified habitats, we used our model to infer biodiversity across the sampled sites, based on the values of the explanatory variables at the time of bumble bee sampling, compared to model predictions under a reference condition in which we assumed that landscapes were entirely composed of natural habitat, with no history of human modification, subject to no application of pesticides, and with no change in climate. Although this reference is entirely hypothetical, not reflecting actual conditions at any time in recent history, it nevertheless serves to illustrate the likely magnitude of human activities on bumble bee assemblages across natural and human‐modified habitats. To derive model‐estimated differences between reference and actual conditions, we drew 1000 sets of coefficient estimates from the posterior samples of the model. We then calculated estimates of the percentage difference in average occurrence probability between reference and actual environmental conditions for each of these sets of coefficient estimates.

## 3 Results

**FIGURE 1:**
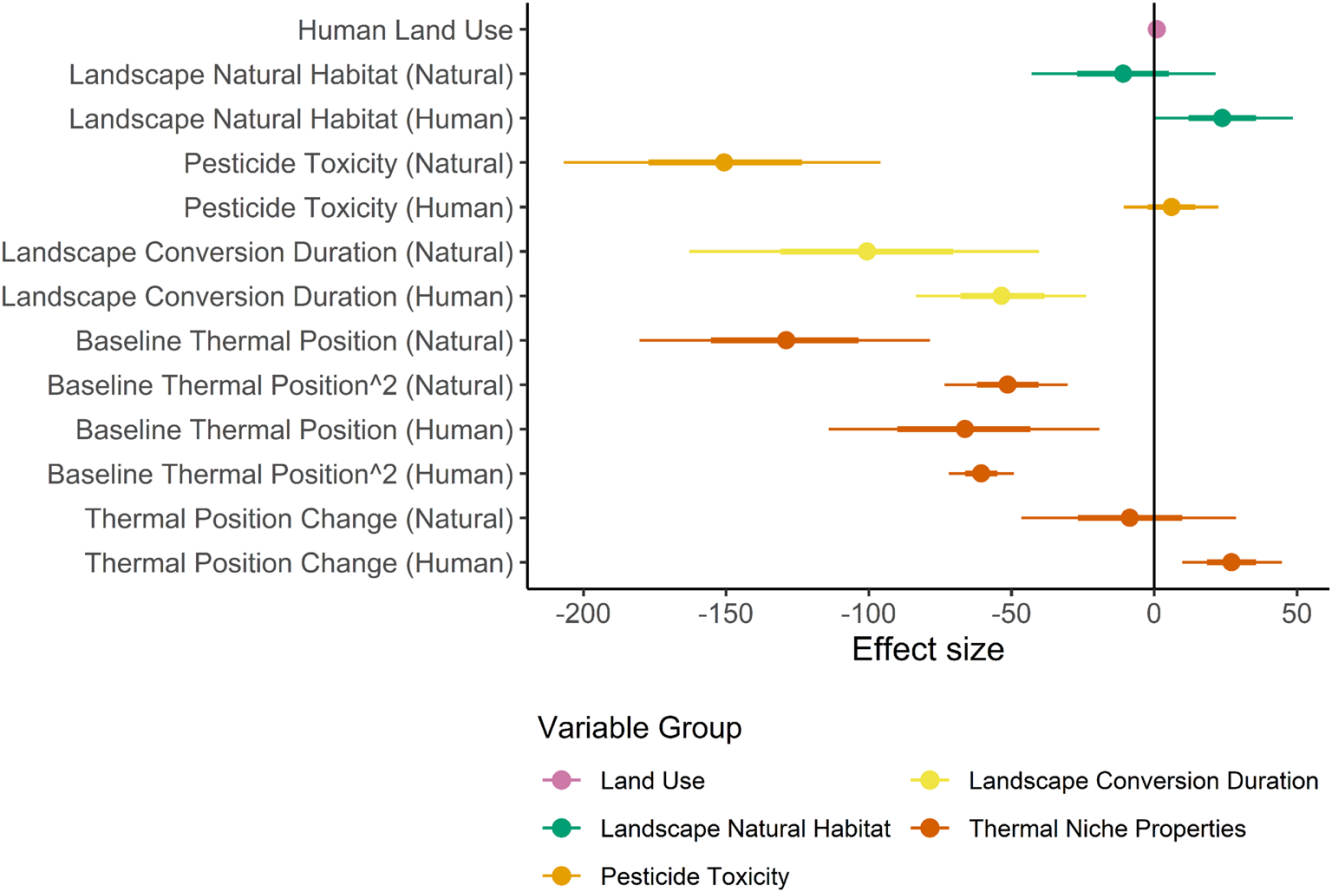
Forest plot of coefficient estimates from the full binomial Bayesian hierarchical model relating bumble bee occurrence to land use, landscape habitat, pesticide toxicity and thermal niche properties. Points represent median coefficient estimates, thick horizontal bars the 67% credible intervals, and thin bars the 95% credible intervals. Effects whose 95% credible intervals do not cross zero are interpreted as being ‘significant’. Coefficient estimates are coloured by variable grouping: Purple—land use; green—surrounding natural habitat; light orange—pesticide toxicity; yellow—duration of substantial landscape human habitat modification; dark orange—realised thermal niche position and effect on this of climate change. Text in parentheses refers to whether the relationship is for sites with natural local habitat or with human land use. For the baseline thermal niche position, the Baseline Thermal Position and Baseline Thermal Position^2 coefficients refer to the linear and quadratic components of the polynomial relationship, respectively, combined to describe the curivlinear relationship shown in Figure 2.

Landscape availability of natural habitat, duration of substantial human habitat modification, cumulative pesticide toxicity, thermal niche properties, and climate change all contributed to explaining bumble bee occurrence probability, with effects strongly dependent on local land‐use type (Figure 1). A model containing just land‐use type explained little variation in bumble bee occurrence (*R* ^2^_marginal_ = 0.004; *R* ^2^_conditional_ = 0.31). The full model was substantially better (*R* ^2^_marginal_ = 0.13; *R* ^2^_conditional_ = 0.32). In the full model, land‐use type had a negligible effect on bumble bee probability of occurrence but played an important role in interaction with the other variables.

**FIGURE 2:**
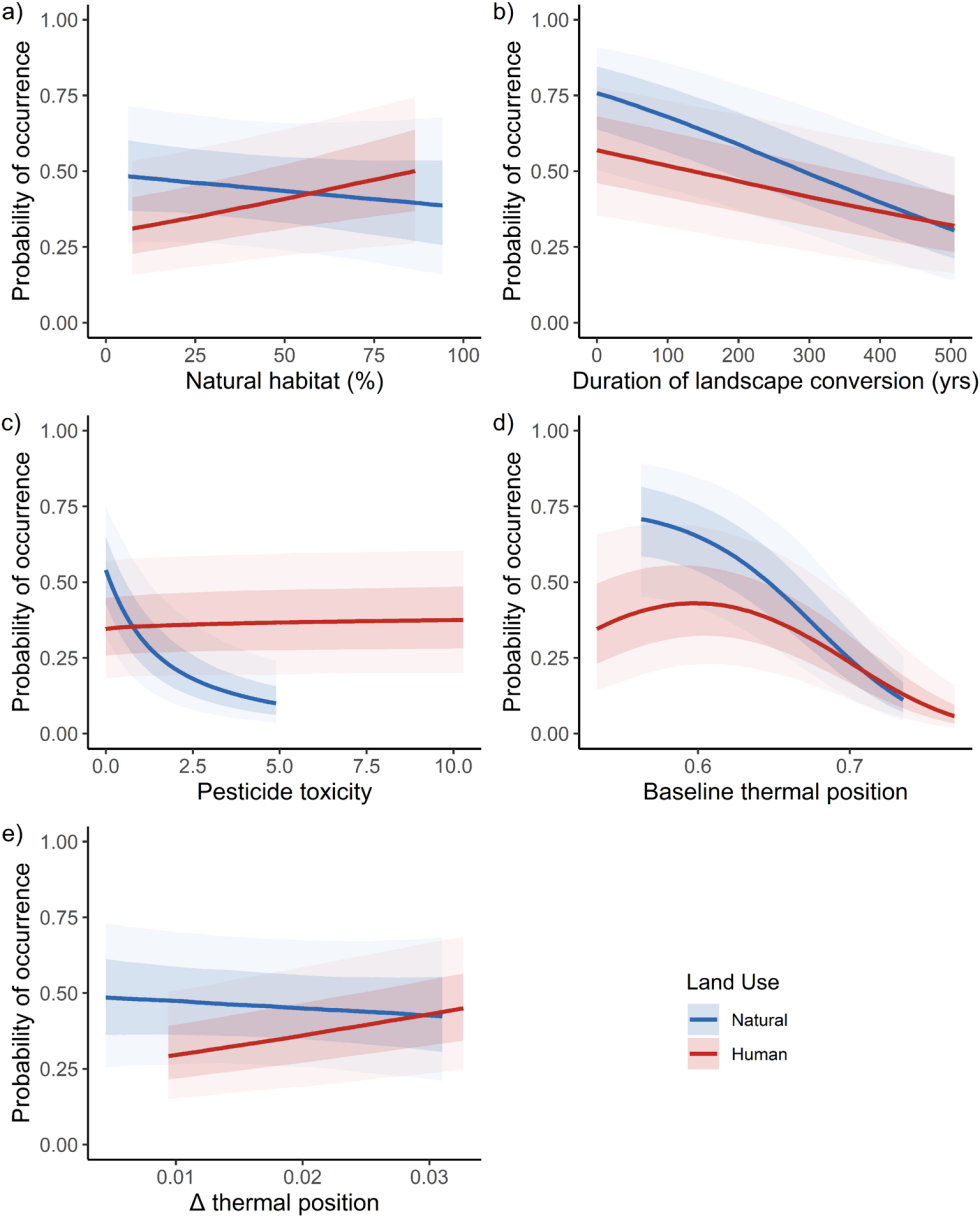
Conditional modelled effects of landscape natural habitat (a), duration of landscape modification (b), pesticide toxicity (c), baseline thermal niche position (d) and change in thermal niche position from climate change (e) on the probability of occurrence of bumble bee species. In all panels, lines represent median estimates of conditional effects, for natural (blue) or human‐modified (red) habitats, more opaque shading (in the same colours) represents the 67% credible intervals, and more transparent shading the 95% credible intervals. Variables not being considered are held at their median values. Relationships are plotted for the central 95% of values sampled within each land use for each explanatory variable.

Landscape natural habitat availability had a significant positive effect on occurrence probability in human‐modified habitats, but no clear effect within natural habitats (Figure 2a). In both natural and human‐modified habitats, bumble bees are less likely to occur in landscapes with a longer duration of substantial human habitat modification (Figure 2b). In natural habitats, the probability of occurrence of bumble bees decreases strongly with pesticide toxicity, whereas in human‐modified habitats there is a negligible effect (Figure 2c).

Populations near the centre of species' thermal niches (the exact centre of the niche has a thermal position of 0.5) showed the greatest reduction in probability of occurrence in human‐modified compared to natural habitats, whereas for populations near the upper thermal niche limit, occurrence probability was similar in natural and human‐modified habitats (Figure 2d). Increases in realised thermal niche position caused by climate warming were associated with a negligible effect on occurrence probability in natural habitats, but a positive effect in human‐modified habitats (Figures 1 and 2e).

Individual model chains converged well (all Rhat ≤ 1; Figure S4). There was a good correspondence between model predicted values and observations (Figure S5). A Moran's test across all model residuals did not reveal significant spatial autocorrelation (*I* = −0.0034; *p* = 0.94). However, there was significant spatial autocorrelation (*p* < 0.05) in the residuals associated with 12.5% of the underlying studies in the dataset, higher than the 5% expected by chance (Figure S6). Importantly, the records from studies with significant residual spatial autocorrelation did not occupy extreme values of any explanatory variable (Figure S3), and so bias in coefficient estimates is unlikely. Results were very similar when landscape natural‐habitat availability was estimated at 1‐ or 5‐km spatial grain (Figures S7 and S8), and when using high instead of low estimates of cumulative pesticide toxicity (Figure S11). Estimates of the effect of duration of substantial habitat modification were weaker (although qualitatively unchanged) when substantial habitat modification was defined using a threshold of 10% or 50% (rather than 30%) conversion of landscapes to human‐modified land uses (Figures S9 and S10). Results were also robust when fitting a zero‐inflated negative binomial model of relative abundance, the key differences being that effects of natural habitat were somewhat weaker, and effects of pesticide toxicity were stronger (Figure S12).

**FIGURE 3:**
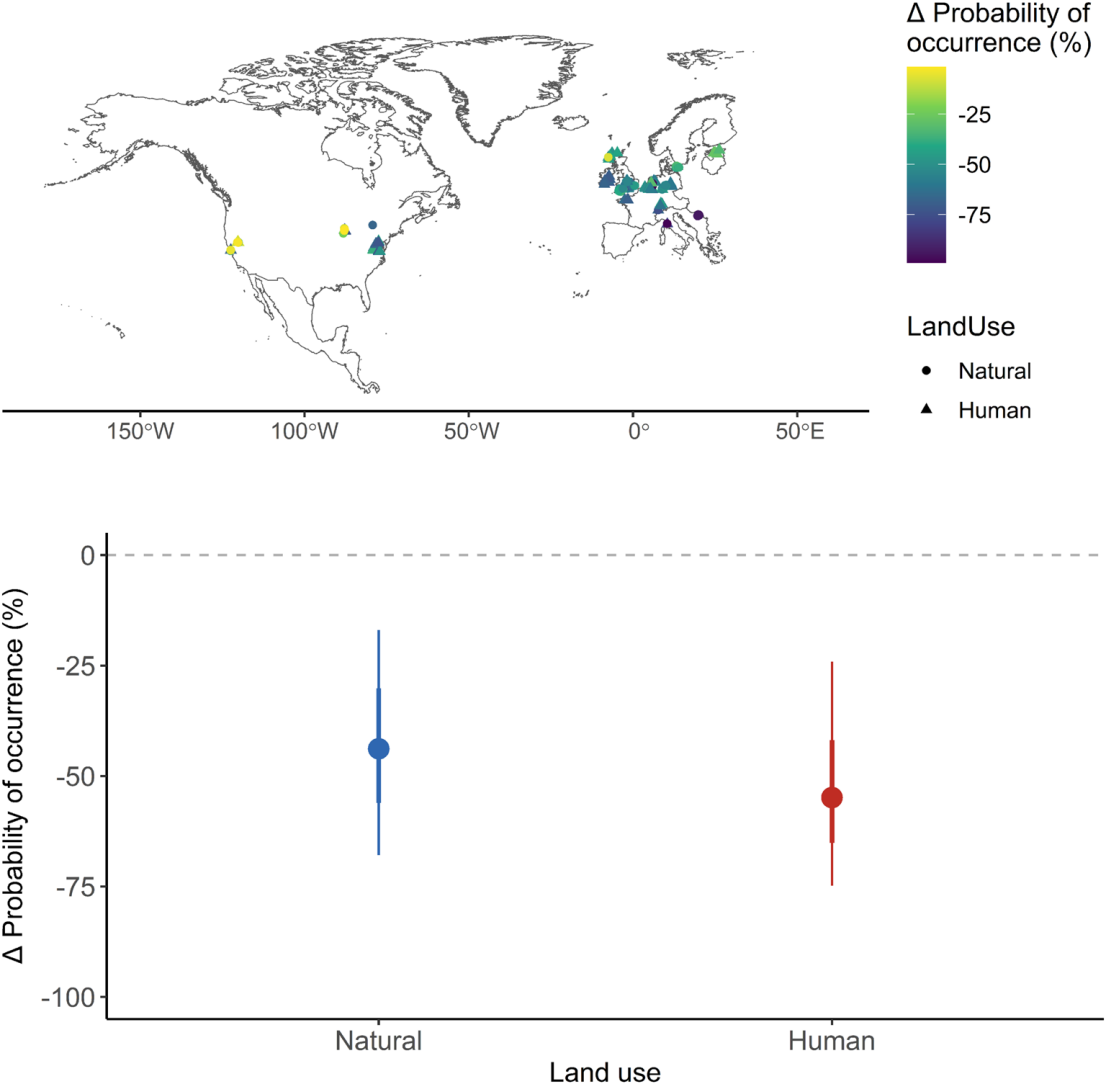
Model‐inferred differences in average bumble bee occurrence probability at sampled sites in natural and human‐modified habitats, compared to hypothetical reference conditions (natural land use across the whole landscape, no human land‐use history, no agricultural chemical application, and no climate change). Predictions were generated for every species recorded at each sampled site across both natural and human‐modified habitats, based on the estimates of the explanatory variables. Points show the median prediction, while thick and thin error bars represent the 67% and 95% confidence intervals, respectively, based on sampling 1000 coefficient estimates from the model posteriors.

Compared to estimated occurrence probability under hypothetical reference conditions (100% surrounding natural habitat, and no history of human land use, application of agricultural chemicals or climate change), we estimate that bumble bee probability of occurrence is lower by 44% (95% CI: 17%–68%) across sampled natural habitats, and by 55% (95% CI: 24%–75%) across sampled human land uses (Figure 3).

## 4 Discussion

Our results reveal important interactive effects of climate change, landscape habitat modification and local land‐use conditions on bumble bee occurrence probability. Climate‐land‐use interactions may exacerbate biodiversity change (Oliver and Morecroft 2014; Williams and Newbold 2020), but relatively few studies have focused on insects (Fourcade et al. 2019; Hamblin et al. 2017; Hemberger and Williams 2024; Janousek et al. 2023; Kammerer et al. 2021; Oliver et al. 2017; Outhwaite et al. 2022; Waldock et al. 2020). We estimate that bumble bee occurrence probability has been reduced substantially across both natural and human‐modified habitats as a result of human pressures.

We find that bumble bee occurrence probability is reduced in human‐modified habitats in landscapes with lower cover of natural habitats, and across all habitats in landscapes that have been substantially modified by humans for a longer time. Our findings are consistent with previous, local or regional‐scale studies, which generally found negative effects of human land use and positive effects of landscape semi‐natural habitat on bumble bees (Janousek et al. 2023; Proesmans et al. 2019; Samuelson et al. 2018). We show the generality of these effects across two north‐temperate continents and show for the first time the importance of landscape land‐use history in shaping responses.

Interestingly, occurrence probability is reduced most by human land use in the centre of species' thermal niches (indicated by a greater difference in occurrence probability between natural and human‐modified habitats; Figure 2d), in contrast with previous research both on vertebrates (Williams and Newbold 2021) and on insects (Waldock et al. 2020). This may arise because the generally higher occurrence frequency near the niche centre means that factors other than climate play an important role in shaping species presence or absence. For example, floral resources are essential for bumble bees (McCombs et al. 2022; Requier et al. 2020), and are known to be limiting in some high‐intensity agricultural landscapes, particularly at certain times of year (Timberlake et al. 2019).

Climate warming, which moves populations closer to species' upper thermal limits, increased occurrence probability in human land uses. This contrasts with the results of a study on birds in North America, where declines in response to habitat loss were strongest where summer temperatures had warmed most (Northrup et al. 2019). This finding may indicate that bumble bee species found within human‐modified habitats constitute a filtered set that are relatively more resilient to intensifying pressures such as climate change. Overall, the interactive effects of climate change and land use appear more complicated for bumble bees than for other groups of species, and it is important to note that varying rates of warming in different locations will add further complexity.

The cumulative toxicity of pesticides applied in landscapes is associated with steep reductions in occurrence probability of bumble bees in natural habitats (often these natural habitats are in close proximity to farmland), but surprisingly not within agricultural areas. The absence of an effect of pesticide toxicity in agricultural areas could again indicate that bumble bee assemblages in these areas are already filtered to include only those species relatively more tolerant of human pressures. Indeed, a study of UK bees found detectable, but weak, effects of pesticide (specifically neonicotinoid) exposure on population trends within farmed landscapes (Woodcock et al. 2016). Nevertheless, we caution that the available global data on application of agricultural chemicals are resolved only at a coarse spatial resolution of approximately 10 km (Maggi et al. 2019; Mueller et al. 2012), and future studies will benefit from more finely resolved estimates of pesticide application (Mesnage et al. 2021).

Using our models to infer changes in bumble bee occurrence probability as a result of cumulative pressures suggests that landscape modification, combined with recent climate change, has reduced bumble bee occurrence probability within natural habitats almost as much as in human‐modified areas. While agricultural areas experience the direct impacts of habitat loss, many natural habitats are exposed to degradation of their surrounding landscapes. Additionally, both natural and human‐modified habitats are exposed to the effects of climate change. Land‐use impacts are often quantified using spatial analyses, comparing sampled biodiversity between natural and modified areas (Newbold et al. 2015). The fact that natural habitats are composed of depauperate communities, heavily impacted by changes in their landscapes, means that such estimates likely underestimate land‐use impacts substantially (Newbold et al. 2019).

Understanding changes in biodiversity through time using a spatial analysis is inevitably subject to limitations. Most importantly, spatial analyses cannot consider potential time‐lagged effects of environmental changes (De Palma et al. 2018). However, time‐series data for insects are lacking in most regions. Second, there is likely important interspecific variation in responses (Hemberger et al. 2021), but the database we used did not have sufficient sampling of individual species to consider this. Third, large‐scale studies typically rely on coarsely resolved estimates of pressures. While the availability of fine‐scale land‐use/land‐cover data has improved rapidly in recent years (Jung et al. 2020), we still lack fine‐scale estimates of pesticide application, a particularly important pressure for flying insects (Wagner et al. 2021). The classification of local land‐use type we used is also thematically coarse, simply dividing sites into natural or human‐modified habitats. Our models revealed relatively little difference overall in probability of occurrence between these land‐use types, although there were important interactive effects with the other explanatory variables. To estimate the position of populations within species' thermal niches, we use information on species' realised distributions with respect to temperature. Such realised temperature limits may not correspond with the fundamental physiological limits of species, but estimates of the latter (Bennett et al. 2018) are available for too few species to be useful in large‐scale analyses. A previous study showed that realised thermal limits are clearly associated with occupancy change in response to recent climate change (Soroye et al. 2020), suggesting these limits are ecologically important. We detected significant spatial autocorrelation in the residuals associated with a slightly higher fraction of studies than expected by chance, which may lead to some uncertainty in the modelled effects, although the fact that studies whose residuals showed significant spatial autocorrelation did not occupy extreme values of any of the explanatory variables suggests that substantial bias is unlikely. In general, correlative analyses can be consistent with, but not prove, causal mechanisms. The complexity of the interactive effects we document highlights a need for experimental studies or detailed field‐level assessments to investigate the mechanistic basis for the patterns we report. Finally, it is important to highlight the entirely hypothetical reference against which we estimated potential changes in bumblebee assemblages caused by human activities. While this reference isn't expected to reflect actual environmental conditions at any time in recent history, it serves to illustrate the potential magnitude of human impacts on bumble bee biodiversity, across both natural and human‐modified habitats.

In conclusion, interactions between climate change, climatic niche position, and land‐use pressures (contemporary and historical) drive substantial changes in bumble bee occurrence across both natural and human‐modified habitats. Given the importance of bumble bees for pollinating wild plants (Ollerton 2017) and agricultural crops (Pritchard and Vallejo‐Marín 2020), these changes are likely to have important effects on both natural ecosystems and on our ability to grow food. Ongoing changes in climate and land use are likely to lead to a further reshaping of bumble bee communities across natural and human‐modified habitats. To predict future changes in biodiversity robustly, including of bumble bees, we must account for the complex interactive effects of climate change and land use.

## Author Contributions

T.N., J.K., P.S. and J.J.W. designed the analyses. T.N. performed all main analyses. T.N. and P.S. provided the methods for quantifying species' thermal niche position. T.N. wrote the first draft of the manuscript. All authors contributed to editing the manuscript. T.N. and J.K. secured the funding to support the presented research.

## Conflicts of Interest

The authors declare no conflicts of interest.

### Peer Review

The peer review history for this article is available at https://www.webofscience.com/api/gateway/wos/peer‐review/10.1111/ele.70145.

## Supporting information


Data S1.

## Data Availability

The code needed to run all of the analyses is publicly available on GitHub (https://github.com/timnewbold/BumblebeesLUClimStudy). The data needed to run the code are published on FigShare, DOI: (10.6084/m9.figshare.28713143), except for datasets for which we do not have permission to republish, but which are freely available in other public repositories. Instructions for obtaining these additional datasets are given both in the FigShare repository description and at the top of the first two code scripts.
