# Supplementary material for: Bumble Bee Probability of Occurrence Responds to Interactions Between Local and Landscape Land Use, Climatic Niche Properties and Climate Change
Source: Ecol Lett. 2025 May 27;28(5):e70145. doi: 10.1111/ele.70145 (PMC12107511; doi:10.1111/ele.70145)
Supplement: Supplementary file 1 — Data S1. [file ELE-28-0-s001.docx]

Supplementary Material for ‘Bumble bee probability of occurrence responds to interactions between local and landscape land use, climatic niche properties and climate change’

Tim Newbold^1,*^, Jeremy Kerr^2^, Peter Soroye^2,†^, Jessica J. Williams^1,‡^

^1^Centre for Biodiversity and Environment Research, Department of Genetics, Evolution and Environment, University College London, London, UK

^2^Department of Biology, University of Ottawa, Ottawa, Ontario, Canada

^†^Current address: Wildlife Conservation Society Canada, Toronto, Ontario, Canada

^‡^Current address: Department of Life Sciences, Silwood Park Campus, Imperial College London, London, UK

^*^Correspondence: [t.newbold@ucl.ac.uk](mailto:t.newbold@ucl.ac.uk)

# Sites in Analysis


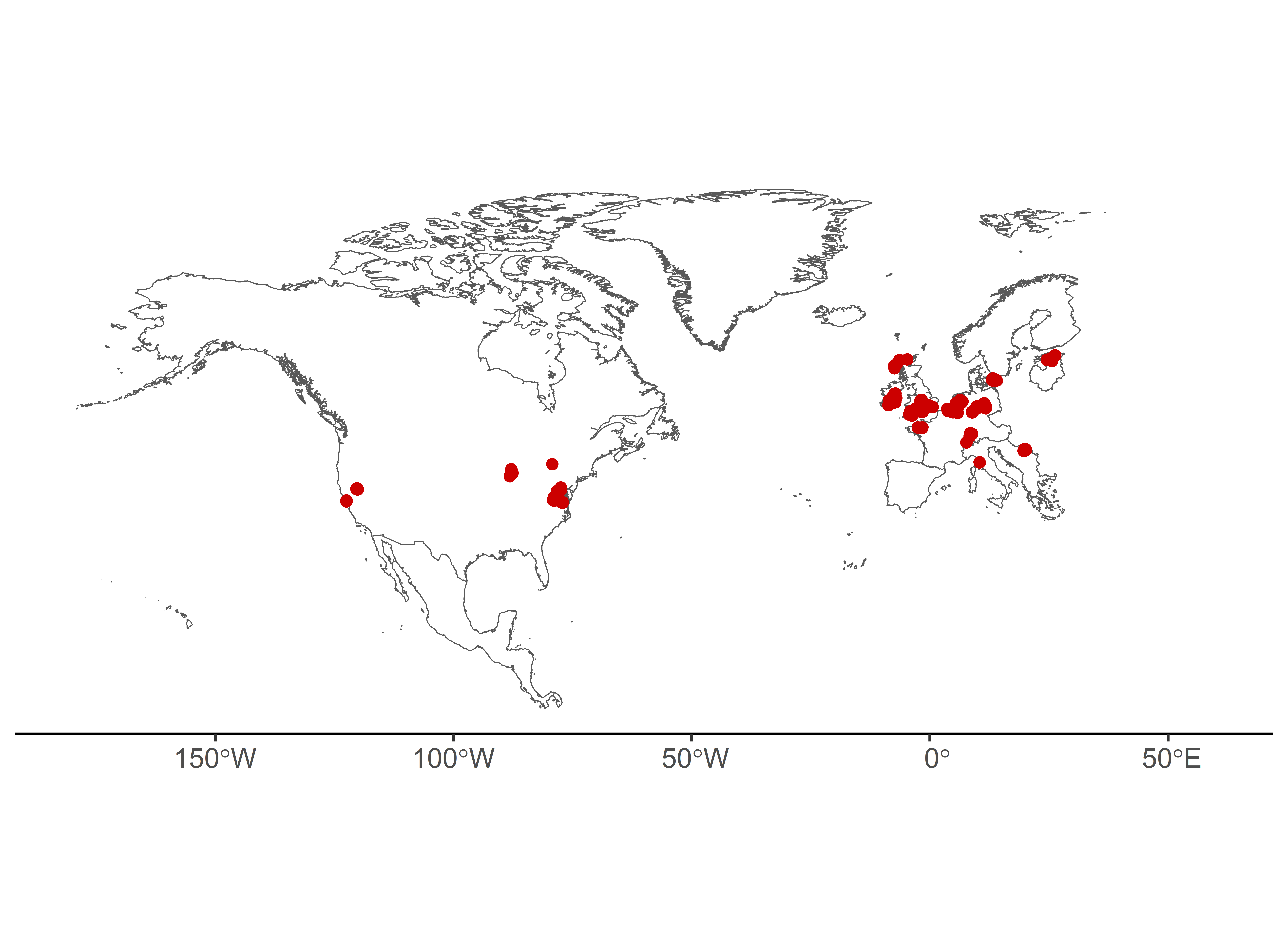


**Figure S1.** **Location of sites used in the analysis**. Base map shows the UN sub-regions from which bumble bee data were obtained: Northern America, Central America, Northern Europe, Western Europe and Southern Europe. The 13 countries sampled were: Canada, the United States, Belgium, Estonia, France, Germany, the Republic of Ireland, Italy, the Netherlands, Serbia, Sweden, Switzerland and the United Kingdom. Note that Mexico is classed within Central America in the UN sub-region scheme. Map is plotted with geographical coordinates using the WGS 1984 datum

# Species in Analysis

**Table S1. List of all species included in the analysis**

| Species |
| --- |
| Bombus auricomus |
| Bombus barbutellus |
| Bombus bifarius |
| Bombus bimaculatus |
| Bombus bohemicus |
| Bombus campestris |
| Bombus centralis |
| Bombus citrinus |
| Bombus cryptarum |
| Bombus distinguendus |
| Bombus fervidus |
| Bombus flavifrons |
| Bombus griseocollis |
| Bombus hortorum |
| Bombus humilis |
| Bombus hypnorum |
| Bombus impatiens |
| Bombus insularis |
| Bombus jonellus |
| Bombus lapidarius |
| Bombus lucorum |
| Bombus magnus |
| Bombus melanopygus |
| Bombus mesomelas |
| Bombus mixtus |
| Bombus monticola |
| Bombus muscorum |
| Bombus nevadensis |
| Bombus norvegicus |
| Bombus occidentalis |
| Bombus pascuorum |
| Bombus pratorum |
| Bombus quadricolor |
| Bombus ruderarius |
| Bombus ruderatus |
| Bombus rufocinctus |
| Bombus rupestris |
| Bombus soroeensis |
| Bombus subterraneus |
| Bombus sylvarum |
| Bombus sylvestris |
| Bombus ternarius |
| Bombus terrestris |
| Bombus vagans |
| Bombus vandykei |
| Bombus vestalis |
| Bombus veteranus |
| Bombus vosnesenskii |
| Bombus wurflenii |


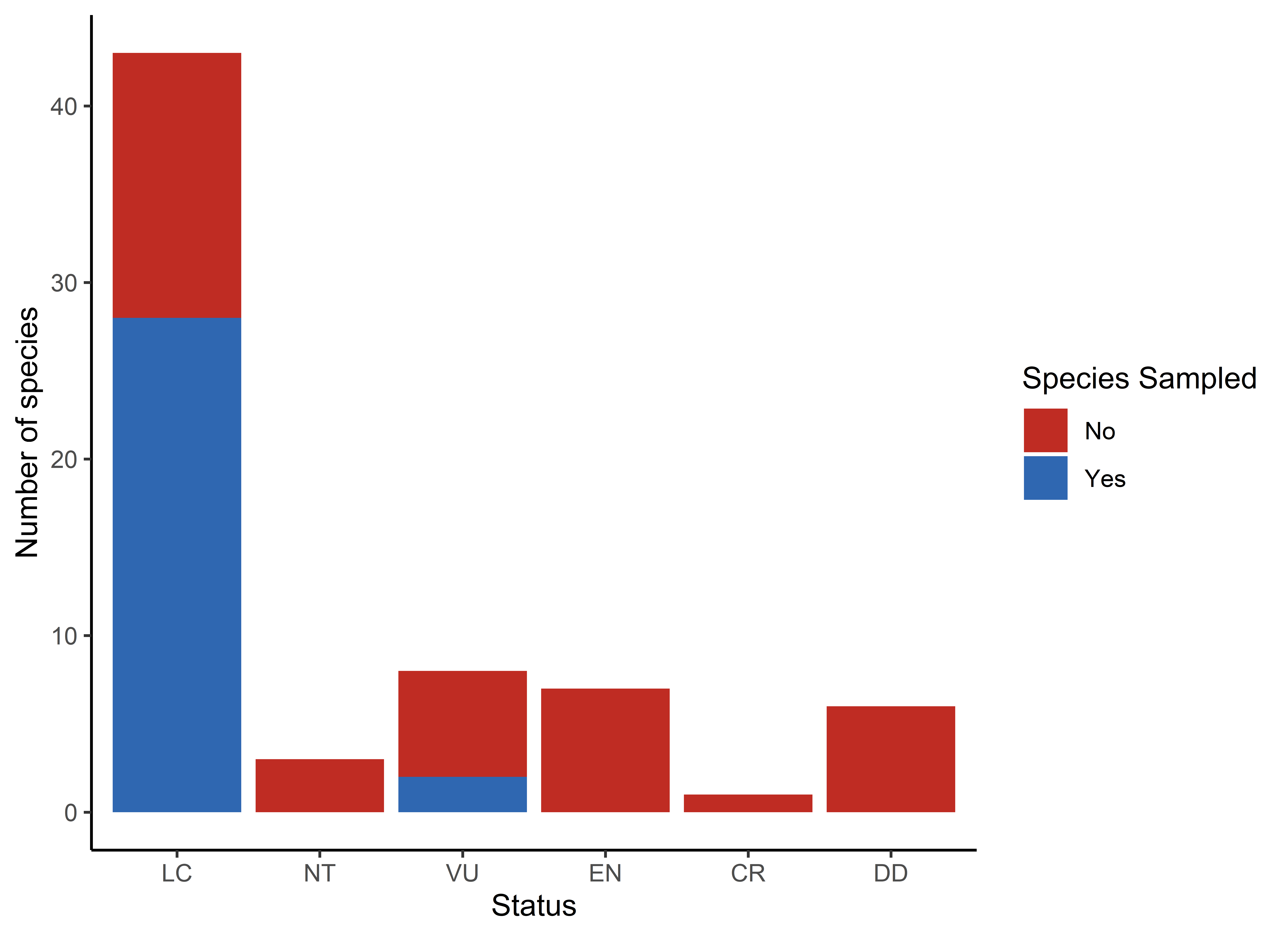


**Figure S2.** **Representation of species with different threat statuses in the model**. For European bumble bee species, for which all species have been assigned a Red List status (<https://portals.iucn.org/library/sites/library/files/documents/RL-4-019.pdf>), the distribution of sampled and unsampled species among different levels of extinction risk (LC = Least Concern, NT = Near Threatened, VU = Vulnerable, EN = Endangered, CR = Critically Endangered, DD = Data Deficient). Reveals a bias in sampling towards species with lower levels of assessed extinction risk.

# Distributions of Explanatory Variables





Figure S3: **Distributions of the records included in the analysis along the gradients of the explanatory variables**. Histograms are plotted separately for natural habitats (blue colour) and human-modified land uses (red colour). Black points indicate the position of records within studies that showed significant residual spatial autocorrelation in the final model

# Model Diagnostic Checks











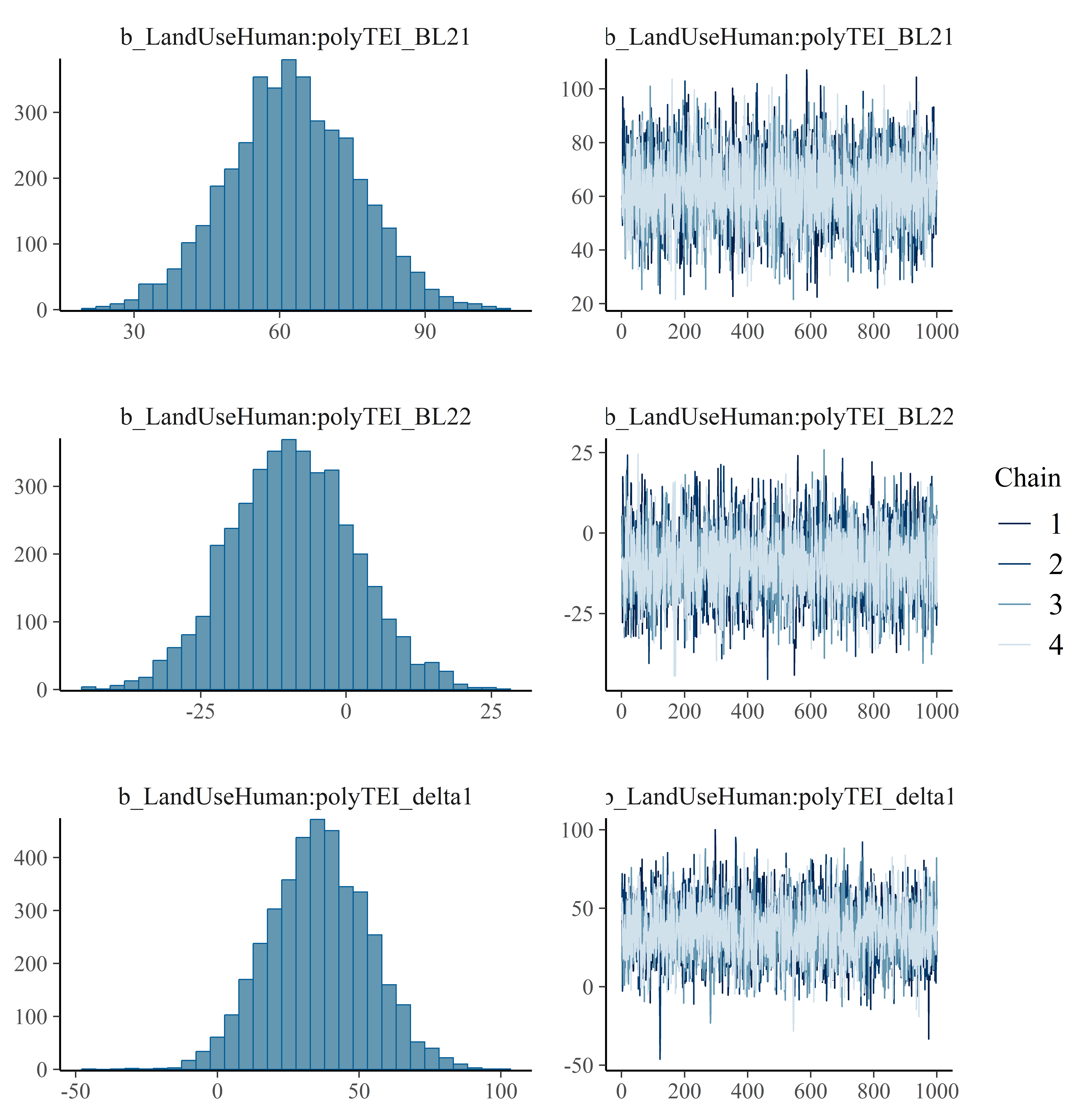


***Figure S4.* *Density and trace plots for model coefficient estimates****. For each modelled parameter, left-hand panels show density plots of coefficient estimates from the posterior distribution, while right-hand panels trace plots for each of four model chains across the post-burn-in sampling period. Plots were produced using the plot function in the brms R package Version 2.21.0.*


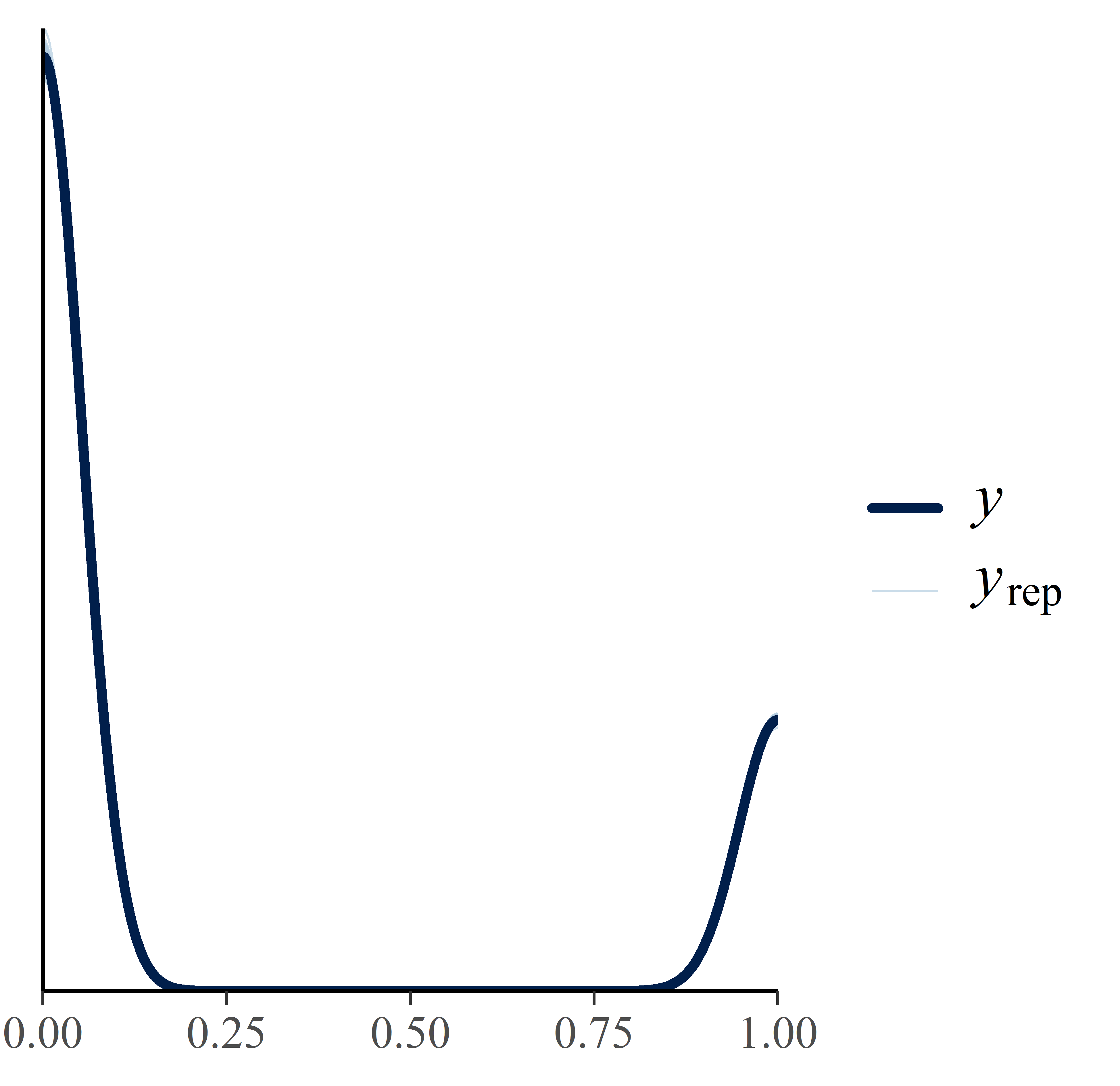


**Figure S5.** **Density plot comparing observed presences/absences with model-predicted values drawn from the posterior distribution**. Figure is produced directly by the pp_check function of the brms R package Version 2.21.0


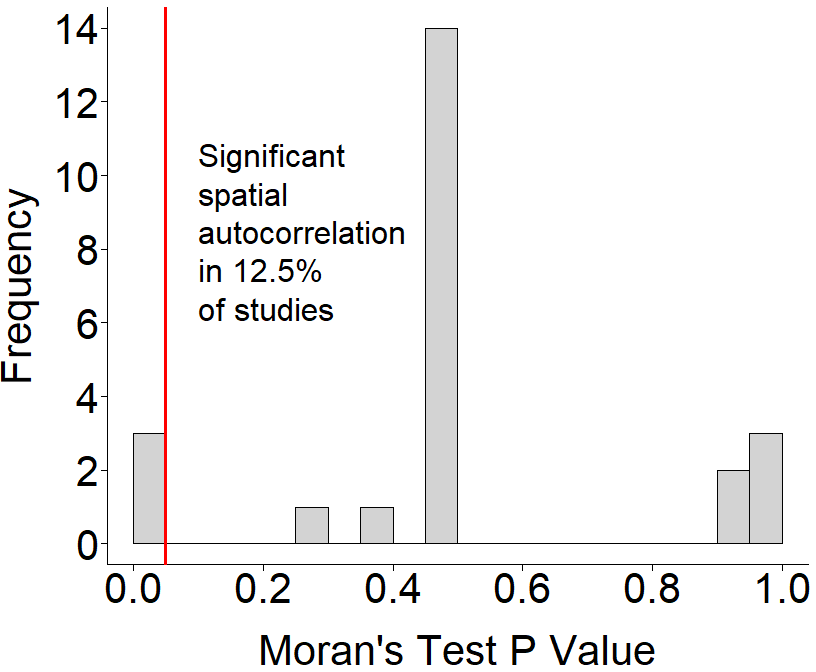


**Figure S6.** **Distribution of P values from a series of Moran’s tests for spatial autocorrelation in the residuals associated with each individual study**. For each study, we calculated the average residual for each sampled site, and then ran a Moran’s test for spatial autocorrelation in these average residuals. Moran’s tests were performed using the spdep R package Version 1.2-8

# Model Robustness Checks


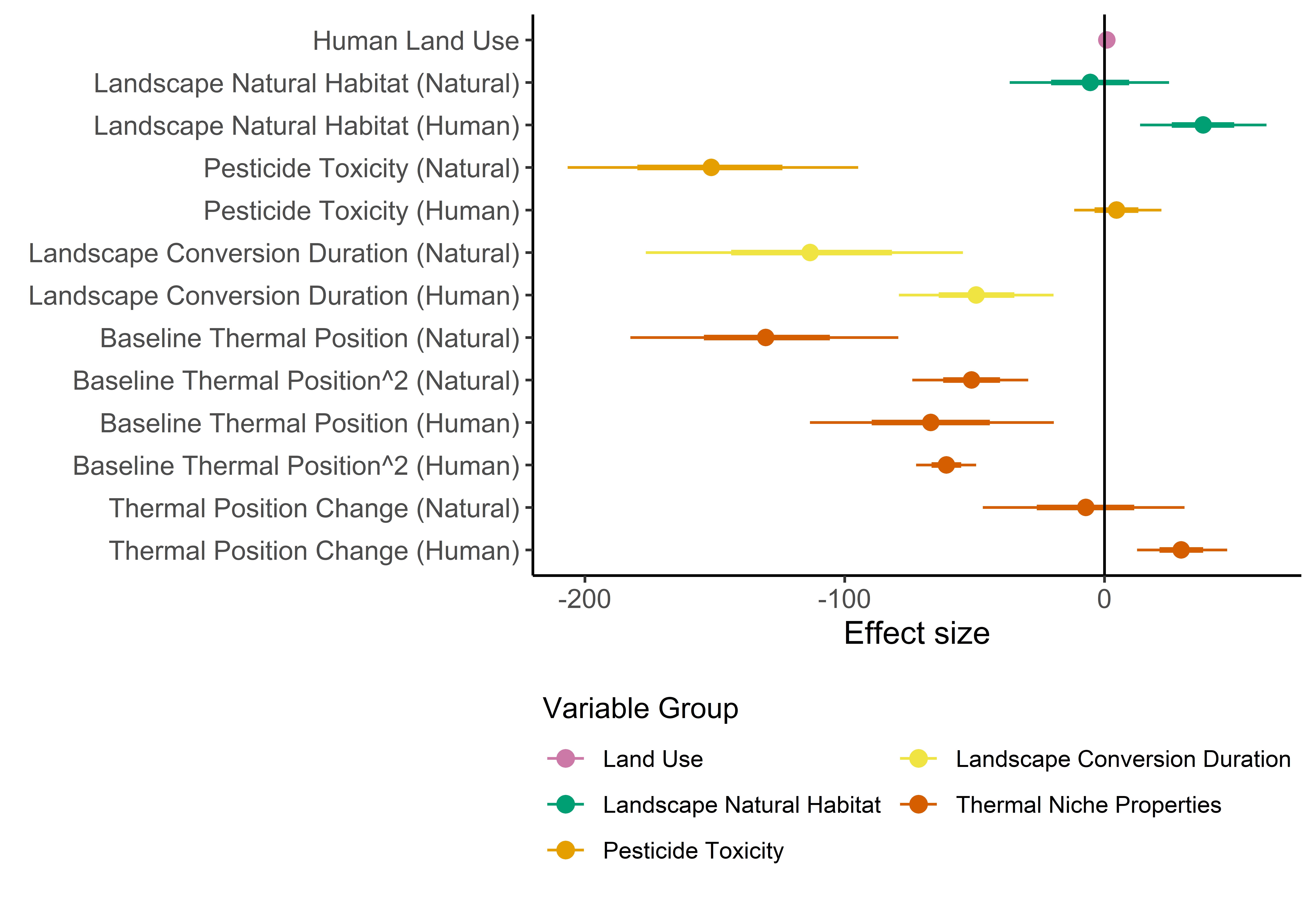


**Figure S7.** **Forest plot of coefficient estimates from the full binomial Bayesian hierarchical model relating bumble bee occurrence to land use, landscape habitat, pesticide toxicity and thermal niche properties**. From the model testing robustness to fitting estimates of landscape natural habitat at 1-km instead of 2-km spatial grain. Points represent median coefficient estimates, thick horizontal bars the 67% credible intervals, and thin bars the 95% credible intervals. Effects whose 95% credible intervals do not cross zero are interpreted as being ‘significant’. Coefficient estimates are coloured by variable grouping: purple - land use; green - landscape natural habitat; light orange - pesticide toxicity; yellow - duration of substantial landscape habitat modification to human uses; dark orange - realized thermal niche position and effect on this of climate change. Text in parentheses refers to whether the relationship is for sites with natural local habitat or with human land use. For the baseline thermal niche position, the Baseline Thermal Position and Baseline Thermal Position^2 coefficients refer to the linear and quadratic components of the polynomial relationship, respectively, combined to describe the curivlinear relationship shown in Figure 2 in the main text.


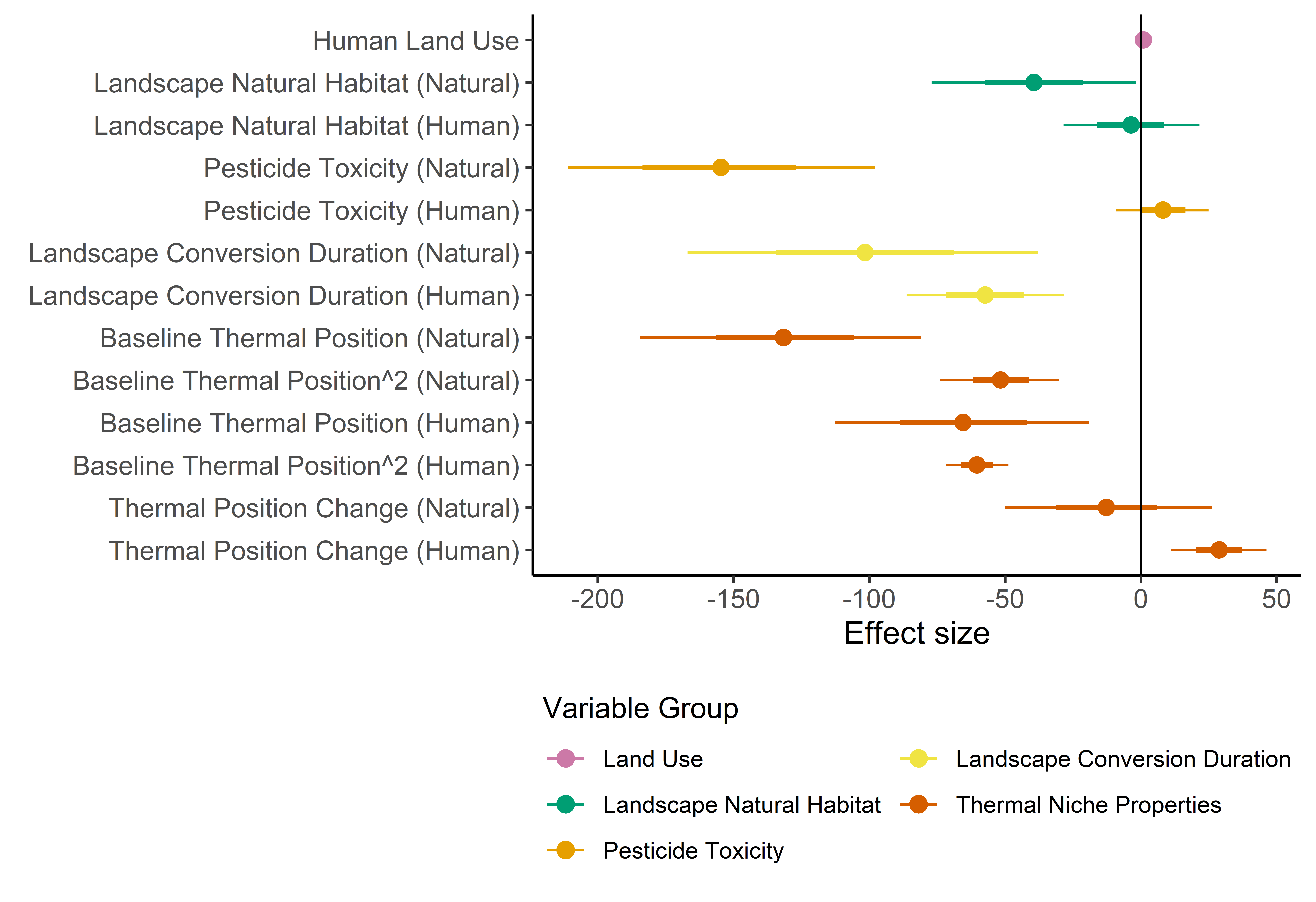


**Figure S8.** **Forest plot of coefficient estimates from the full binomial Bayesian hierarchical model relating bumble bee occurrence to land use, landscape habitat, pesticide toxicity and thermal niche properties**. From the model testing robustness to fitting estimates of landscape natural habitat at 5-km instead of 2-km spatial grain. Points represent median coefficient estimates, thick horizontal bars the 67% credible intervals, and thin bars the 95% credible intervals. Effects whose 95% credible intervals do not cross zero are interpreted as being ‘significant’. Coefficient estimates are coloured by variable grouping: purple - land use; green - landscape natural habitat; light orange - pesticide toxicity; yellow - duration of substantial landscape habitat modification to human uses; dark orange - realized thermal niche position and effect on this of climate change. Text in parentheses refers to whether the relationship is for sites with natural local habitat or with human land use. For the baseline thermal niche position, the Baseline Thermal Position and Baseline Thermal Position^2 coefficients refer to the linear and quadratic components of the polynomial relationship, respectively, combined to describe the curivlinear relationship shown in Figure 2 in the main text.


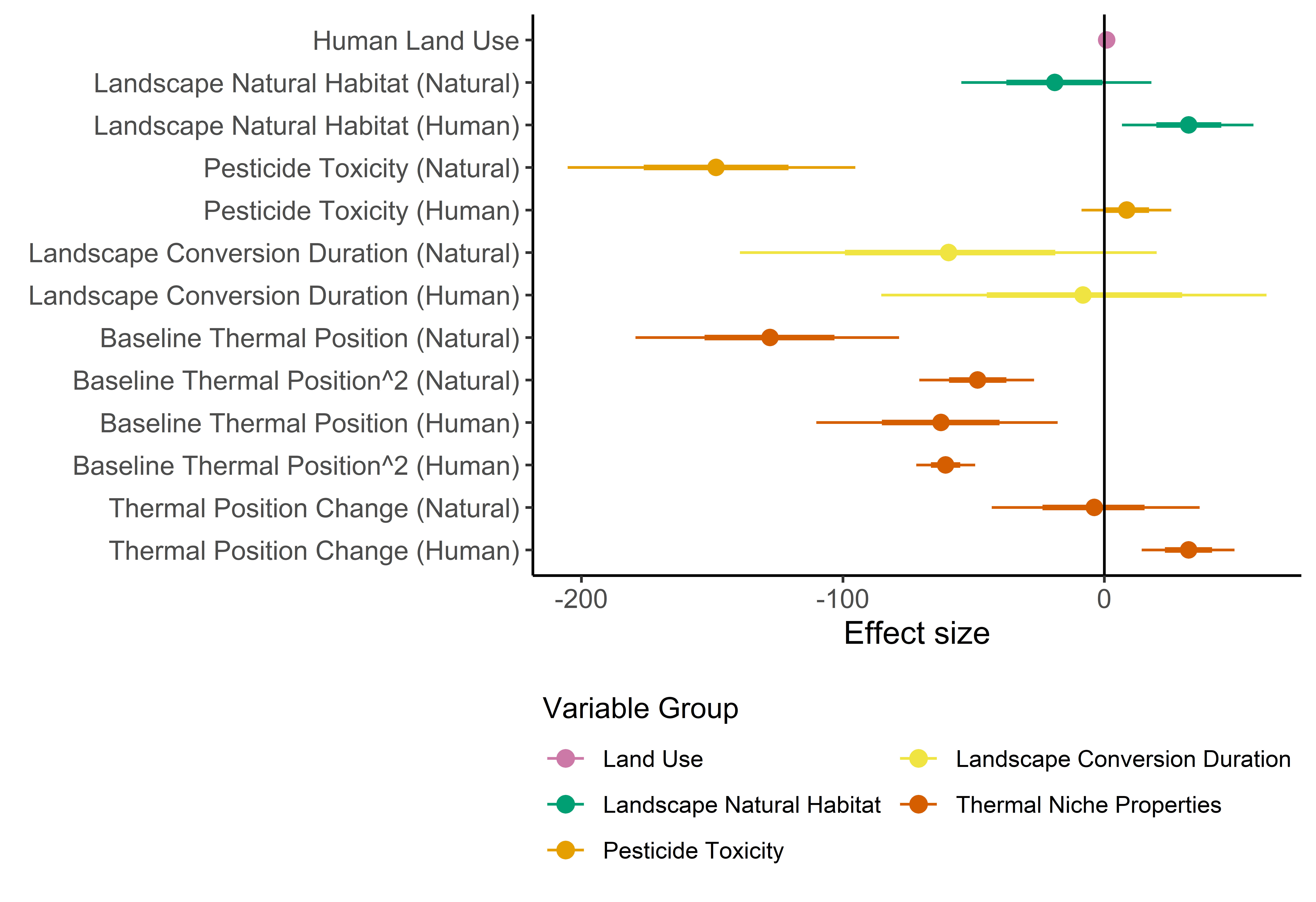


**Figure S9.** **Forest plot of coefficient estimates from the full binomial Bayesian hierarchical model relating bumble bee occurrence to land use, landscape habitat, pesticide toxicity and thermal niche properties**. From the model testing robustness to fitting estimates of duration of substantial habitat modification where the threshold for defining substantial habitat modification is set at 10% instead of 30%. Points represent median coefficient estimates, thick horizontal bars the 67% credible intervals, and thin bars the 95% credible intervals. Effects whose 95% credible intervals do not cross zero are interpreted as being ‘significant’. Coefficient estimates are coloured by variable grouping: purple - land use; green - landscape natural habitat; light orange - pesticide toxicity; yellow - duration of substantial landscape habitat modification to human uses; dark orange - realized thermal niche position and effect on this of climate change. Text in parentheses refers to whether the relationship is for sites with natural local habitat or with human land use. For the baseline thermal niche position, the Baseline Thermal Position and Baseline Thermal Position^2 coefficients refer to the linear and quadratic components of the polynomial relationship, respectively, combined to describe the curivlinear relationship shown in Figure 2 in the main text.


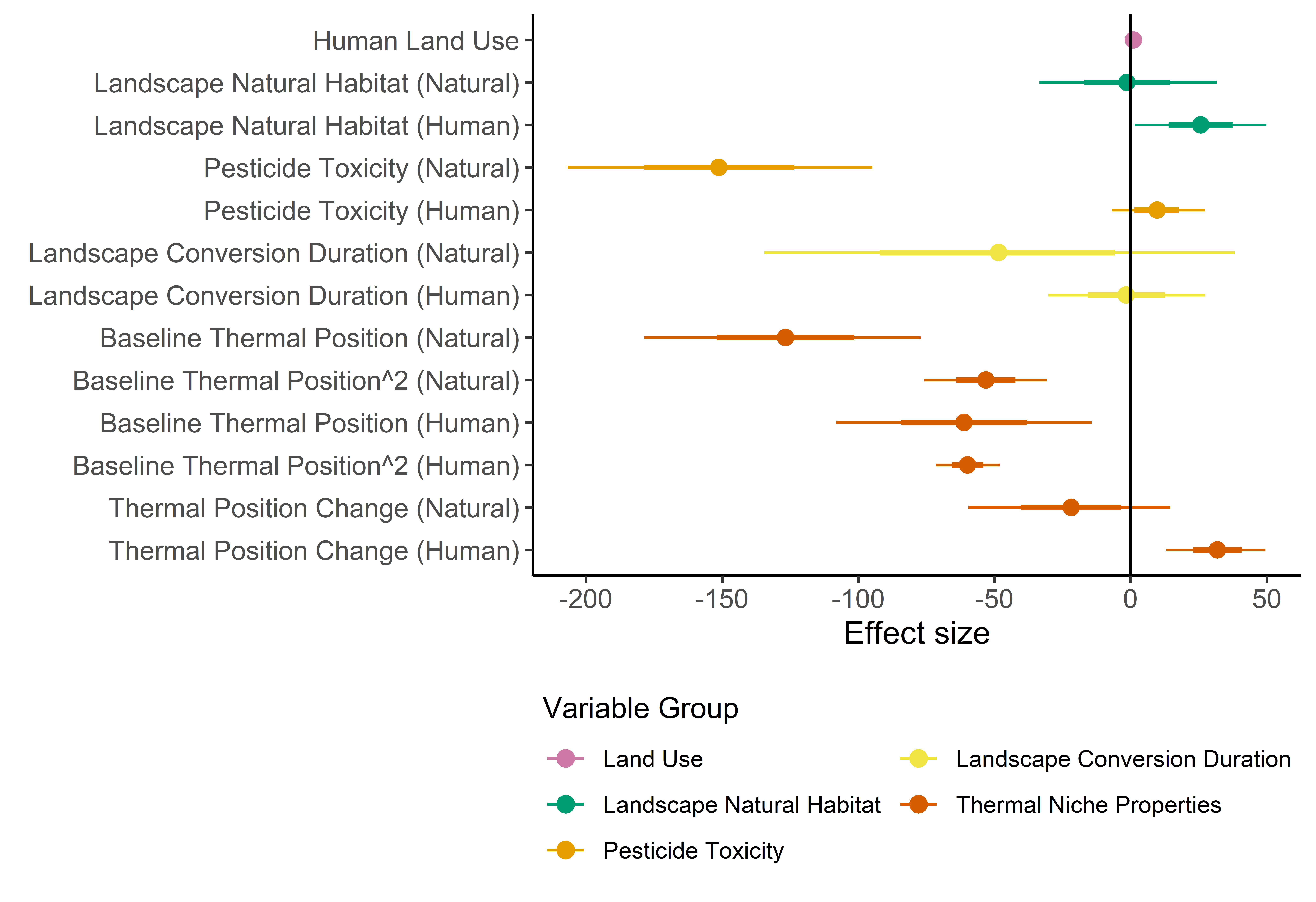


**Figure S10.** **Forest plot of coefficient estimates from the full binomial Bayesian hierarchical model relating bumble bee occurrence to land use, landscape habitat, pesticide toxicity and thermal niche properties**. From the model testing robustness to fitting estimates of duration of substantial habitat modification where the threshold for defining substantial habitat modification is set at 50% instead of 30%. Points represent median coefficient estimates, thick horizontal bars the 67% credible intervals, and thin bars the 95% credible intervals. Effects whose 95% credible intervals do not cross zero are interpreted as being ‘significant’. Coefficient estimates are coloured by variable grouping: purple - land use; green - landscape natural habitat; light orange - pesticide toxicity; yellow - duration of substantial landscape habitat modification to human uses; dark orange - realized thermal niche position and effect on this of climate change. Text in parentheses refers to whether the relationship is for sites with natural local habitat or with human land use. For the baseline thermal niche position, the Baseline Thermal Position and Baseline Thermal Position^2 coefficients refer to the linear and quadratic components of the polynomial relationship, respectively, combined to describe the curivlinear relationship shown in Figure 2 in the main text.


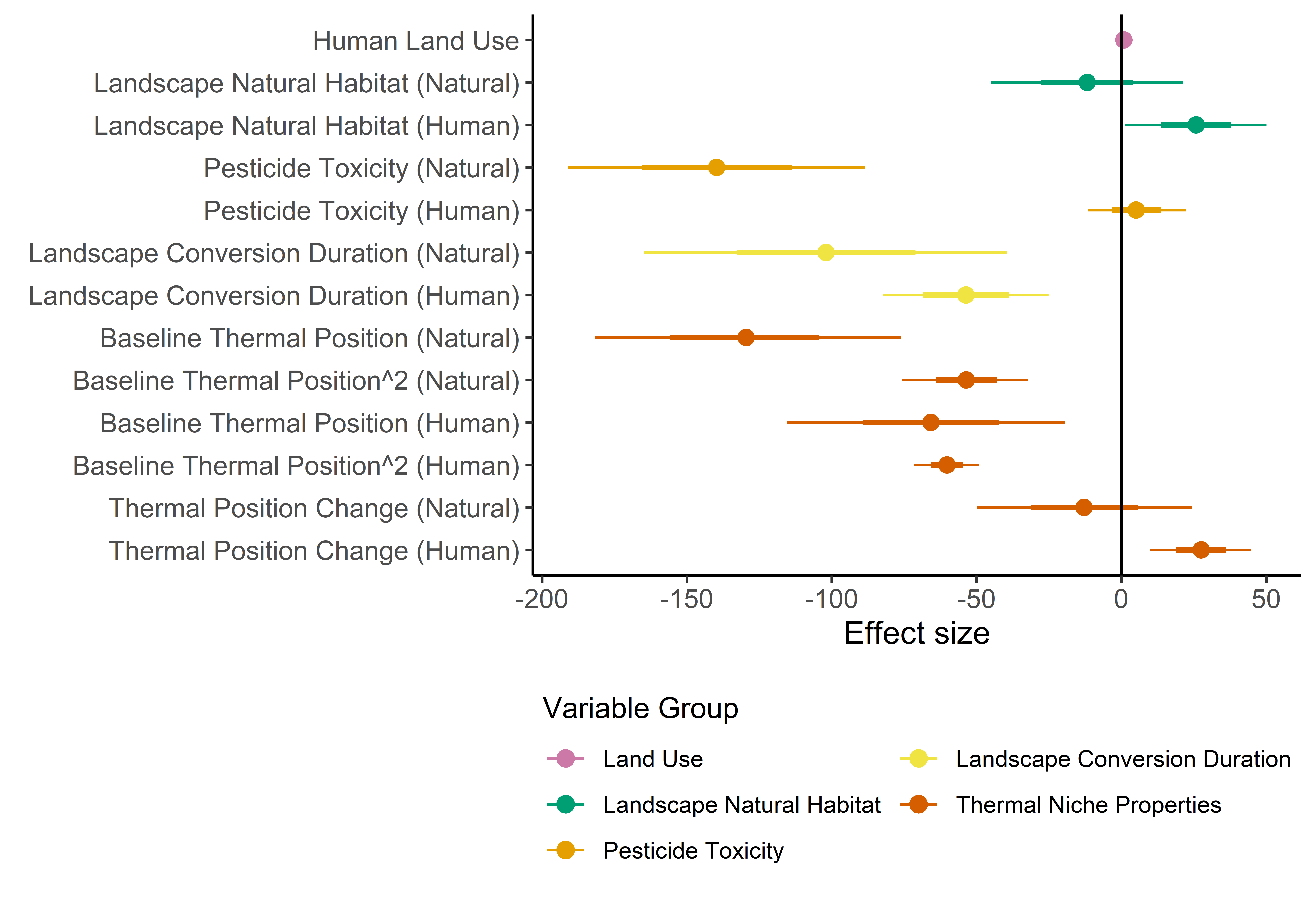


**Figure S11.** **Forest plot of coefficient estimates from the full binomial Bayesian hierarchical model relating bumble bee occurrence to land use, landscape habitat, pesticide toxicity and thermal niche properties**. From the model testing robustness to using high instead of low estimates of pesticide application density. Points represent median coefficient estimates, thick horizontal bars the 67% credible intervals, and thin bars the 95% credible intervals. Effects whose 95% credible intervals do not cross zero are interpreted as being ‘significant’. Coefficient estimates are coloured by variable grouping: purple - land use; green - landscape natural habitat; light orange - pesticide toxicity; yellow - duration of substantial landscape habitat modification to human uses; dark orange - realized thermal niche position and effect on this of climate change. Text in parentheses refers to whether the relationship is for sites with natural local habitat or with human land use. For the baseline thermal niche position, the Baseline Thermal Position and Baseline Thermal Position^2 coefficients refer to the linear and quadratic components of the polynomial relationship, respectively, combined to describe the curivlinear relationship shown in Figure 2 in the main text.


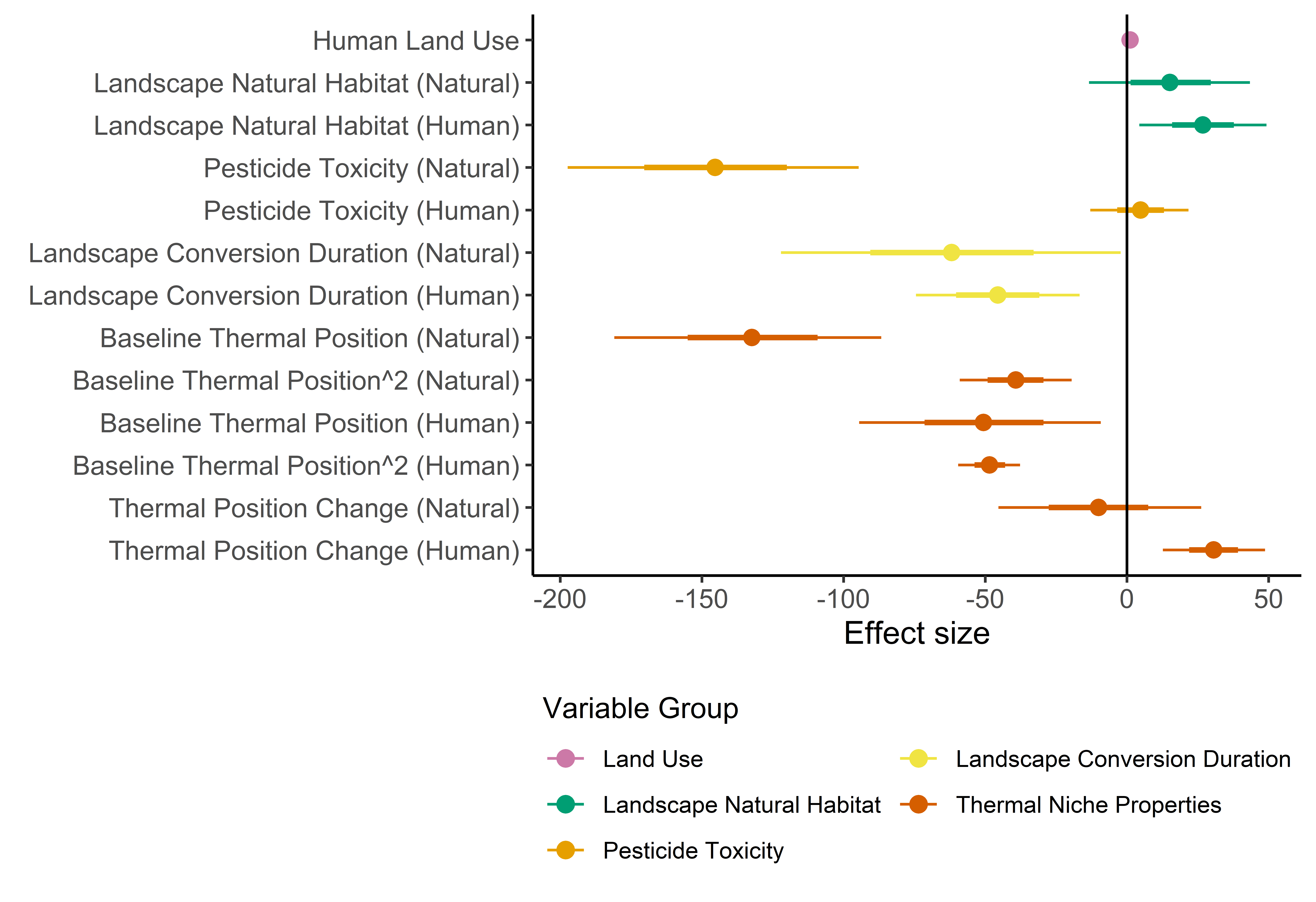


**Figure S12.** **Forest plot of coefficient estimates from a zero-inflated negative binomial model relating bumble bee relative abundance to land use, landscape habitat, pesticide toxicity and thermal niche properties**. Points represent median coefficient estimates, thick horizontal bars the 67% credible intervals, and thin bars the 95% credible intervals. Effects whose 95% credible intervals do not cross zero are interpreted as being ‘significant’. Coefficient estimates are coloured by variable grouping: purple - land use; green - landscape natural habitat; light orange - pesticide toxicity; yellow - duration of substantial landscape habitat modification to human uses; dark orange - realized thermal niche position and effect on this of climate change. Text in parentheses refers to whether the relationship is for sites with natural local habitat or with human land use. For the baseline thermal niche position, the Baseline Thermal Position and Baseline Thermal Position^2 coefficients refer to the linear and quadratic components of the polynomial relationship, respectively, combined to describe the curivlinear relationship shown in Figure 2 in the main text.

# References to Original Data Papers in PREDICTS Database

Bates, A.J., Sadler, J.P., Fairbrass, A.J., Falk, S.J., Hale, J.D. & Matthews, T.J. (2011). [Changing bee and hoverfly pollinator assemblages along an urban-rural gradient](https://doi.org/10.1371/journal.pone.0023459). *PLoS ONE*, 6, e23459.

Billeter, R., Liira, J., Bailey, D., Bugter, R., Arens, P., Augenstein, I., *et al.* (2007). [Indicators for biodiversity in agricultural landscapes: A pan‐European study](https://doi.org/10.1111/j.1365-2664.2007.01393.x). *Journal of Applied Ecology*, 45, 141–150.

Blake, R.J., Westbury, D.B., Woodcock, B.A., Sutton, P. & Potts, S.G. (2011). [Enhancing habitat to help the plight of the bumblebee](https://doi.org/10.1002/ps.2136). *Pest Management Science*, 67, 377–379.

Connop, S., Hill, T., Steer, J. & Shaw, P. (2010). [Microsatellite analysis reveals the spatial dynamics of *Bombus humilis* and *Bombus sylvarum*: Spatial dynamics of foraging bumblebees](https://doi.org/10.1111/j.1752-4598.2010.00116.x). *Insect Conservation and Diversity*, 4, 212–221.

Darvill, B., Knight, M.E. & Goulson, D. (2004). [Use of genetic markers to quantify bumblebee foraging range and nest density](https://doi.org/10.1111/j.0030-1299.2004.13510.x). *Oikos*, 107, 471–478.

Diekötter, T., Walther-Hellwig, K., Conradi, M., Suter, M. & Frankl, R. (2006). [Effects of landscape elements on the distribution of the rare bumblebee species *Bombus muscorum* in an agricultural landscape](https://doi.org/10.1007/s10531-004-2932-9). *Biodiversity and Conservation*, 15, 57–68.

Fowler, R.E. (2014). *An investigation into bee assemblage change along an urban-rural gradient*. PhD thesis. University of Birmingham.

Goulson, D., Lepais, O., O’Connor, S., Osborne, J.L., Sanderson, R.A., Cussans, J., *et al.* (2010). [Effects of land use at a landscape scale on bumblebee nest density and survival](https://doi.org/10.1111/j.1365-2664.2010.01872.x). *Journal of Applied Ecology*, 47, 1207–1215.

Hanley, M.E., Franco, M., Dean, C.E., Franklin, E.L., Harris, H.R., Haynes, A.G., *et al.* (2011). [Increased bumblebee abundance along the margins of a mass flowering crop: Evidence for pollinator spill‐over](https://doi.org/10.1111/j.1600-0706.2011.19233.x). *Oikos*, 120, 1618–1624.

Hatfield, R.G. & LeBuhn, G. (2007). [Patch and landscape factors shape community assemblage of bumble bees, *Bombus* spp. (Hymenoptera: Apidae), in montane meadows](https://doi.org/10.1016/j.biocon.2007.06.019). *Biological Conservation*, 139, 150–158.

Hermann, F., Westphal, C., Moritz, R.F.A. & Steffan‐Dewenter, I. (2007). [Genetic diversity and mass resources promote colony size and forager densities of a social bee (*Bombus pascuorum*) in agricultural landscapes](https://doi.org/10.1111/j.1365-294x.2007.03226.x). *Molecular Ecology*, 16, 1167–1178.

Jauker, B., Krauss, J., Jauker, F. & Steffan-Dewenter, I. (2012). [Linking life history traits to pollinator loss in fragmented calcareous grasslands](https://doi.org/10.1007/s10980-012-9820-6). *Landscape Ecology*, 28, 107–120.

Julier, H.E. & Roulston, T.H. (2009). [Wild bee abundance and pollination service in cultivated pumpkins: Farm management, nesting behavior and landscape effects](https://doi.org/10.1603/029.102.0214). *Journal of Economic Entomology*, 102, 563–573.

Knight, M.E., Osborne, J.L., Sanderson, R.A., Hale, R.J., Martin, A.P. & Goulson, D. (2009). [Bumblebee nest density and the scale of available forage in arable landscapes](https://doi.org/10.1111/j.1752-4598.2009.00049.x). *Insect Conservation and Diversity*, 2, 116–124.

Kohler, F., Verhulst, J., van Klink, R. & Kleijn, D. (2007). [At what spatial scale do high‐quality habitats enhance the diversity of forbs and pollinators in intensively farmed landscapes?](https://doi.org/10.1111/j.1365-2664.2007.01394.x) *Journal of Applied Ecology*, 45, 753–762.

Le Féon, V., Schermann-Legionnet, A., Delettre, Y., Aviron, S., Billeter, R., Bugter, R., *et al.* (2010). [Intensification of agriculture, landscape composition and wild bee communities: A large scale study in four European countries](https://doi.org/10.1016/j.agee.2010.01.015). *Agriculture, Ecosystems &amp; Environment*, 137, 143–150.

Marshall, E.J.P., West, T.M. & Kleijn, D. (2006). [Impacts of an agri-environment field margin prescription on the flora and fauna of arable farmland in different landscapes](https://doi.org/10.1016/j.agee.2005.08.036). *Agriculture, Ecosystems & Environment*, 113, 36–44.

McFrederick, Q.S. & LeBuhn, G. (2006). [Are urban parks refuges for bumble bees *Bombus* spp. (Hymenoptera: Apidae)?](https://doi.org/10.1016/j.biocon.2005.11.004) *Biological Conservation*, 129, 372–382.

Meyer, B., Gaebele, V. & Steffan-Dewenter, I.D. (2007). Patch size and landscape effects on pollinators and seed set of the horseshoe vetch, *Hippocrepis comosa*, in an agricultural landscape of central Europe. *Entomologia Generalis*, 30, 173–185.

Meyer, B., Jauker, F. & Steffan-Dewenter, I. (2009). [Contrasting resource-dependent responses of hoverfly richness and density to landscape structure](https://doi.org/10.1016/j.baae.2008.01.001). *Basic and Applied Ecology*, 10, 178–186.

Mudri-Stojnic, S., Andric, A., Józan, Z. & Vujic, A. (2012). [Pollinator diversity (Hymenoptera and Diptera) in semi-natural habitats in Serbia during summer](https://doi.org/10.2298/abs1202777s). *Arhiv za Bioloske Nauke*, 64, 777–786.

Oertli, S., Müller, A., Steiner, D., Breitenstein, A. & Dorn, S. (2005). [Cross-taxon congruence of species diversity and community similarity among three insect taxa in a mosaic landscape](https://doi.org/10.1016/j.biocon.2005.05.014). *Biological Conservation*, 126, 195–205.

Osgathorpe, L.M., Park, K. & Goulson, D. (2011). [The use of off-farm habitats by foraging bumblebees in agricultural landscapes: Implications for conservation management](https://doi.org/10.1007/s13592-011-0083-z). *Apidologie*, 43, 113–127.

Power, E.F. & Stout, J.C. (2011). [Organic dairy farming: Impacts on insect-flower interaction networks and pollination: Organic grasslands and pollinators](https://doi.org/10.1111/j.1365-2664.2010.01949.x). *Journal of Applied Ecology*, 48, 561–569.

Quaranta, M., Ambroselli, S., Barro, P., Bella, S., Carini, Alfredo, Celli, G., *et al.* (2004). Wild bees in agroecosystems and semi-natural landscapes. 1997-2000 collection period in Italy. *Bulletin of Insectology*, 57, 11–61.

Redpath, N., Osgathorpe, L.M., Park, K. & Goulson, D. (2010). [Crofting and bumblebee conservation: The impact of land management practices on bumblebee populations in northwest Scotland](https://doi.org/10.1016/j.biocon.2009.11.019). *Biological Conservation*, 143, 492–500.

Richards, M.H., Rutgers-Kelly, A., Gibbs, J., Vickruck, J.L., Rehan, S.M. & Sheffield, C.S. (2011). [Bee diversity in naturalizing patches of carolinian grasslands in southern Ontario, Canada](https://doi.org/10.4039/n11-010). *The Canadian Entomologist*, 143, 279–299.

Samnegård, U., Persson, A.S. & Smith, H.G. (2011). [Gardens benefit bees and enhance pollination in intensively managed farmland](https://doi.org/10.1016/j.biocon.2011.07.008). *Biological Conservation*, 144, 2602–2606.

Tonietto, R., Fant, J., Ascher, J., Ellis, K. & Larkin, D. (2011). [A comparison of bee communities of Chicago green roofs, parks and prairies](https://doi.org/10.1016/j.landurbplan.2011.07.004). *Landscape and Urban Planning*, 103, 102–108.

Verboven, H.A.F., Brys, R. & Hermy, M. (2012). [Sex in the city: Reproductive success of *Digitalis purpurea* in a gradient from urban to rural sites](https://doi.org/10.1016/j.landurbplan.2012.02.015). *Landscape and Urban Planning*, 106, 158–164.
